# Twenty-Year Trajectory-Patterns of Percentage Energy From Dietary Fat vs. Carbohydrate Throughout Adult Life and Associations With Cardio-Metabolic Disease and All-Cause Mortality

**DOI:** 10.3389/fnut.2021.701188

**Published:** 2021-09-06

**Authors:** Xiaoyu Guo, Xiaoqing Xu, Jian Gao, Weiqi Wang, Wanying Hou, Xiaoyan Wu, Changhao Sun, Ying Li, Tianshu Han

**Affiliations:** National Key Discipline, Department of Nutrition and Food Hygiene, School of Public Health, Harbin Medical University, Harbin, China

**Keywords:** twenty-year trajectory-patterns, fat-to-energy ratio, carbohydrate-to-energy ratio, cardio-metabolic disease, mortality

## Abstract

**Background:** The health impacts of dietary fat-to-energy ratio (FER) compared to carbohydrate-to-energy ratio (CER) are widely discussed topics in public health. This study aimed to assess the health impacts of FER and CER by establishing trajectory-patterns of FER and CER over the course of adult life.

**Methods:** This study used the weighted longitudinal data of the China Health and Nutrition Survey, including eight surveys from 1991 to 2011. The trajectories of FER and CER were determined via latent class trajectory modeling. The trajectories were then cross-grouped into different trajectory-patterns. Multivariate Cox regression models were used to assess the relationship between these trajectory-patterns and cardio-metabolic diseases and all-cause mortality. Ten thousand nine hundred and twenty-six adults with a total of 50,693 observations across eight surveys were included.

**Results:** Compared to the trajectory-pattern of persistently low-FER (increased from 10 to 20%) and moderate-CER (stable and ranging from 55 to 65%) over the adult life-course, the two trajectory-patterns that showed changing to high-FER and low-CER were significantly associated with obesity [HR 1.83 [95% CI, 1.10–3.04]; HR 1.46 [95% CI, 1.02–2.17]], diabetes [HR 1.80 [95% CI, 1.03–3.16]; HR 1.49 [95% CI, 1.01–2.25]], and all-cause mortality [HR 2.29 [95% CI, 1.35–3.87]; HR 1.62 [95% CI, 1.18–2.22]]. In contrast, the trajectory-pattern of a persistently low-FER and high-CER diet was not associated with obesity [HR 1.19 [95% CI, 0.82–1.17]], diabetes [HR 1.41 [95% CI, 0.98–2.02]], cardiovascular-disease [HR 1.48 [95% CI, 0.91–2.39]], and all-cause mortality [HR 1.23 [95% CI, 0.94–1.61]].

**Conclusions:** This study indicates that changing to a high-FER and low-CER diet over the course of adult life was significantly associated with obesity, diabetes, and all-cause mortality in the Chinese adult population. In addition, low-FER and high-CER were not associated with cardio-metabolic disease and all-cause mortality. These observations may provide insights into nutritional policy and dietary guidelines.

## Introduction

The health impacts of dietary fat-to-energy ratio (FER) compared to carbohydrate-to-energy ratio (CER) are widely discussed issues in public health. FER should not exceed 30% based on worldwide dietary recommendations (1, 2). However, US dietary guidelines (2015) abandoned the upper limit of FER because there was evidence that replacing total fat with carbohydrates did not reduce the risk of cardiovascular disease (CVD) (3). In contrast, an 18-country prospective study indicated that high FER was related to lower total mortality, while high CER was associated with higher mortality risk (4). A meta-analysis of seven multinational studies came to the opposite conclusion: “Low CER replaced by high FER or high CER were associated with higher total mortality” (5).

With an increasing number of studies supporting opposing directions, the effect of other environmental factors on diet has been largely overlooked (6, 7). At the same time, population-level dietary transition is occurring globally (8–11), especially in developing countries in recent decades (12–14). This leads to a variation in individuals' FER and CER throughout the course of their adult life. Most observational studies have used the single-time point method to assess FER and CER, which cannot reflect dietary intake in the real world. Although a few studies have adopted the cumulative intake method to assess FER and CER across multiple measurements (15, 16) and have demonstrated that it has more advantages than the single-time point (17), this aggregation cannot detect intake variation over time. Establishing trajectories of FER and CER, therefore, can describe intake variations throughout adult life, reflecting the real-world intake of FER and CER. This can provide insights into health impacts and help in establishing dietary guidelines and nutritional policy.

In China, dietary fat intake is increasing faster than in other parts of the world due to dietary transition in parallel with modernization, urbanization, and economic development (18). This presents a unique model for dietary composition change and sufficient variation in FER and CER trajectories. In this study, latent class trajectory modeling (LCTM) was used to characterize FER and CER trajectories over more than 20 years, based on weighted longitudinal data from China. This study aimed to examine the association of FER and CER trajectories with cardio-metabolic disease and all-cause mortality over the adult life.

## Methods

### Ethics Approval

The Institutional Review Committees of the University of North Carolina at Chapel Hill (NC, USA) and the China National Institute of Nutrition and Food Safety at the Chinese Center for Disease Control and Prevention (Beijing, China) approved the survey protocols, instruments, and the process of obtaining informed consent. All participants provided written informed consent before the surveys.

### The China Health and Nutrition Survey

The China Health and Nutrition Survey (CHNS) is a nationwide survey designed to investigate the state of health and nutrition in China. It reflects age, sex, and education profiles (19). Participants were drawn from 228 communities of nine diverse provinces, including eight surveys across 20 years (1991–2011). The provinces in the CHNS represented 47% of the Chinese population based on the 2010 census. Each survey maintained the desired range of economic and demographic variables. It was difficult to calculate response rates and attrition in each survey, however, since some participants who lost to follow-up in one survey year may have participated in subsequent surveys (19).

### Study Population

Our study included adults aged 18–60 years in eight surveys undertaken between 1991 and 2011. By the end of 2011, 32,134 participants provided information on dietary fat and carbohydrate intake. The following individuals were excluded: 473 participants with extreme daily energy intake (<500 or >3,500 kcal/d for women and <800 or >4,200 kcal/d for men); 12,695 participants who were <18 or more than 60 years old; 1,899 participants who were diagnosed with cardio-metabolic diseases in their first survey, and 6,141 participants who participated in the survey only once. The number of visits ranged from two to eight measurement surveys (two visits, *n* = 2,660; three visits, *n* = 1,398; four visits, *n* = 1,482; five visits, *n* = 1,218; six visits, *n* = 1,481; seven visits, *n* = 1,221; eight visits, *n* = 1,466; median = 4.6 visits; total *N* = 10,926 participants across 50,693 observations).

### Dietary Survey

Dietary assessment was based on a combination of three consecutive days of detailed at-home food consumption information via a weighing technique. All food consumed outside the home was also recorded during the 3 days based on 24-h recall. Trained interviewers recorded the types, amounts, dining time, and places for each food intake using photographs and food models. The amount of food in each dish was estimated based on the household inventory and each participant reported the proportion of each dish. The quality of the dietary survey was ensured by the following: (i) All staff took part in a high-quality training course for at least 3 days before the collection of dietary data; and (ii) well-trained interviewers compared an individual's average daily dietary intake with the household inventory. Interviewers revisited households and individuals to further enquire about the individual's food consumption if there were substantial differences. The Chinese Food Composition Table was used to calculate the dietary intake of macronutrients (20).

### Outcome Measures

The outcomes were cardio-metabolic disease, including obesity, diabetes, hypertension, CVD, and all-cause mortality. A portable SECA stadiometer (SECA, Hamburg, Germany) was used to measure individuals' height at each survey (without shoes to the nearest 0.2 cm). A calibrated beam scale was used to measure weight without shoes and in light clothing to the nearest 0.1 kg. Body mass index (BMI) was calculated, and obesity was defined as BMI ≥ 28 kg/m^2^ (21, 22). A total of 10,820 participants provided weight and height, and 977 obesity cases were reported. Diabetes was defined by self-report of a history of diabetes diagnosis, and/or fasting blood glucose ≥ 7.0 mmol/l, and/or HbA1c ≥ 40 mmol/mol (6.5%), and/or receiving diabetes treatment. Only blood samples were used to diagnose diabetes in the 2009 survey. A total of 9,951 participants provided information on diabetes, and 612 diabetes cases were identified. Hypertension was defined by self-report of a history of hypertension diagnosis, and/or systolic blood pressure ≥140 mm Hg, and/or diastolic blood pressure ≥90 mm Hg, and/or receiving treatment for hypertension. A total of 10,887 participants provided blood pressure information, and 3,605 hypertension cases were identified. CVD was defined by self-report of a history of stroke, and/or myocardial infarction. A total of 9,965 participants provided information on CVD, and 240 CVD cases were detected. All-cause mortality status was based on the information documented during the survey. A total of 10,926 participants provided information on mortality, and 632 mortality cases were identified.

### Covariates

There were three education level variables: none, less than high school, and high school or above. The mean amount of alcohol consumption was categorized as none, light (<7 standard drinks per week), moderate (7–14 standard drinks per week), or heavy (>21 standard drinks per week). Smoking was categorized as never, former, or current smoker. The mean of the individual income among the surveys collected as continuous variables was categorized as quartiles. The mean of the total metabolic equivalents (METs) of occupational activity and home activity among the surveys was calculated as MET-h per week (23). Medical insurance was categorized as none, rural, or city. The mean value of BMI was expressed as a continuous variable. The mean intake of food and nutrients was expressed as continuous variables, and included whole-grain, fruit, vegetable, energy, fiber, saturated fatty acid, monounsaturated fatty acid, and polyunsaturated fatty acid. The mean urbanicity index was defined as a continuous variable using a multidimensional 12-component urbanization index capturing community-level physical, social, cultural, and economic environments (24). All mean values were within-individual mean.

### Statistical Analysis

**Figure 1:**
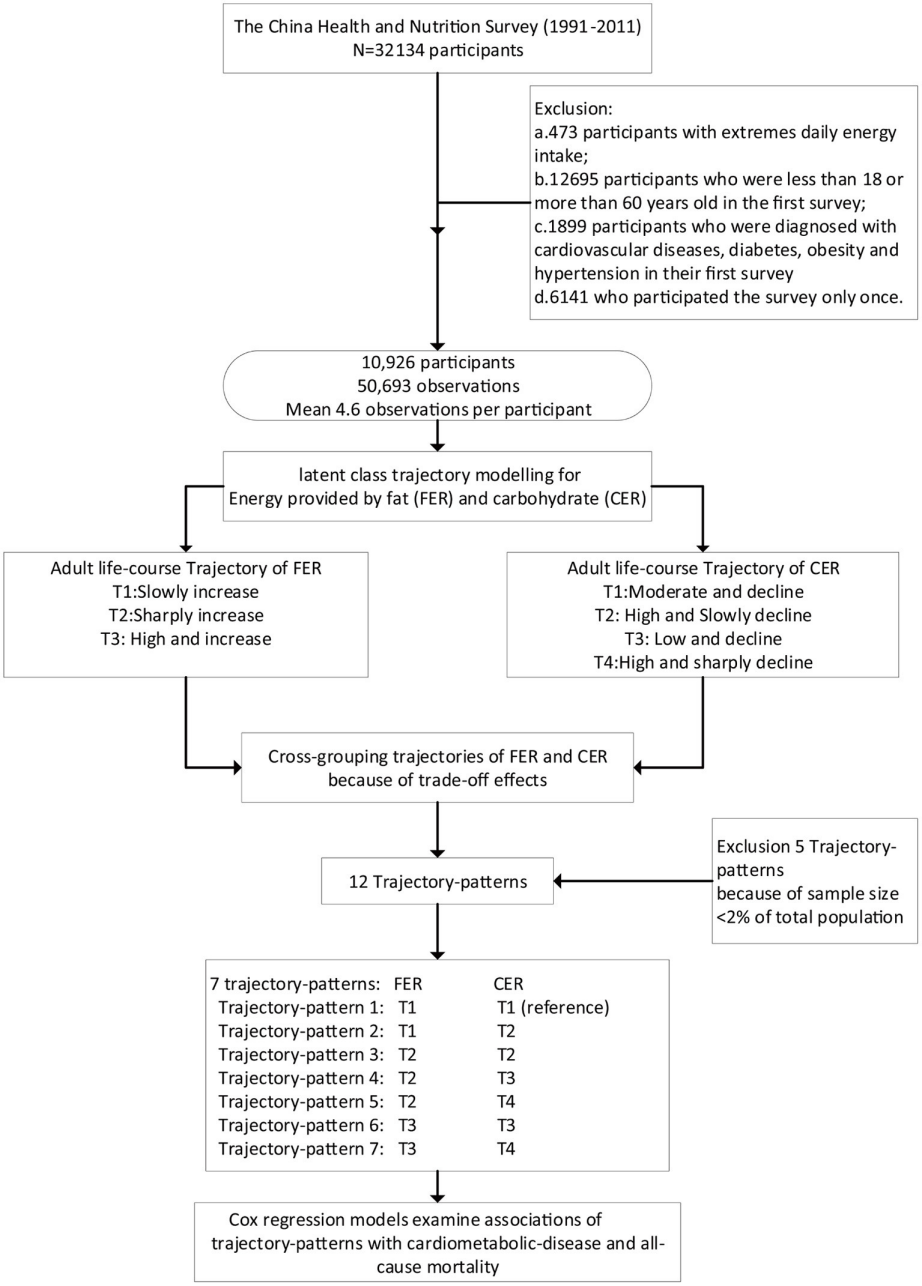
Flow paradigm for this study.

R 3.5.2 (www.r-project.org/) was used for all statistical analyses. Continuous variables were described as means (SDs), while categorical variables were expressed as numbers (percentages). A two-sided *P*-value <0.05 was considered statistically significant. Percentage of energy provided by fat or carbohydrate was determined by dividing energy from the fat or carbohydrate by the total daily energy intake and expressed as FER and CER [FER = [[fat (g) × 9]/total energy intake [kcal]] × 100, CER = [[carbohydrate (g) × 4]/total energy intake [kcal]] × 100]. The Tukey transformation was used to normalize FER and CER to improve the normality of the distribution. The flow diagram of the statistical analysis is shown in Figure 1.

Latent class trajectory modeling (LCTM) was used to identify FER and CER trajectories using the R package lcmm with a censored normal model, with various order polynomials (25). Statistically rigorous criteria were used to determine the best fit (i) using the lowest Bayesian information criterion, and (ii) at least 2% of the sample population within each trajectory class. Once trajectories of dietary FER and CER were determined, two nominal categorical variables were created to describe the FER and CER trajectory classes of each participant. A new variable—‘trajectory-patterns’—was created by cross-grouping trajectories of FER and CER since the ‘trade-off’ effect frequently occurs between FER and CER as, when fat consumption is decreased, intake of carbohydrates often goes up to make up for the decreased calories and vice versa. Twelve trajectory-patterns were created. However, 259 participants were excluded because the sample size was <2% of the total population. These included 20 obesity cases, 26 diabetes cases, 148 hypertension cases, 5 CVD cases, and 23 deaths in the five trajectory-patterns. The remaining seven trajectory-patterns were then used in generalized linear models and Cox regression models.

Generalized linear models were performed to test differences in baseline characteristics of continuous variables across trajectory-patterns with adjustment for age and sex. χ^2^-test was used to measure differences in baseline characteristics of dichotomous variables. Cox regression models were used to estimate the relationship between trajectory-patterns and the risk of cardio-metabolic disease and all-cause mortality. The FER of the reference group selected as the trajectory-pattern increased from 10 to 20%, and CER was stable at between 55 and 65% throughout adulthood. HRs and 95% CI were calculated. Five models were performed: (i) crude model; (ii) model with further adjustment for demographic covariates, including age, sex, smoking status, alcohol consumption status, education level, the mean value of METs, and individual income; (iii) model with further adjustment for nutritional covariates, including the mean value of energy intake, fruit, vegetable, whole-grain, saturated fat, monounsaturated fat, polyunsaturated fat; (iv) model with further adjustment for the mean value of BMI (except when the outcome was obesity); (v) model with further adjustment for social environment covariates, including urbanization index, provinces, and medical insurance.

### Sensitivity Analysis

Three sensitivity analyses were also conducted. The first sensitivity analysis was used to examine whether the ‘sick-quitter’ effect could influence results. The ‘sick-quitter’ effect is based on the assumption that participants may change their FER and CER after being diagnosed with cardio-metabolic disease before the study visit. To account for this, the ratio between FER and CER was calculated, and the joint longitudinal latent class model with time-to-event was used to identify trajectories of the ratio. The trajectories were then automatically linked with the outcomes via the survival rate curve. The second and third sensitivity analyses were to test whether analysis of trajectory-pattern could provide more information than analyses of a single measure or cumulative FER and CER.

## Results

### Participant Characteristics

Characteristics of the study population from the CHNS based on survey years are presented in Supplementary Table 1. Age, BMI, fruit, urbanicity index, and individual income showed increasing trends across survey years. In contrast, total energy intake and carbohydrate intake showed decreasing trends.

### Trajectories of FER and CER Over the Course of Adult Life

**Figure 2:**
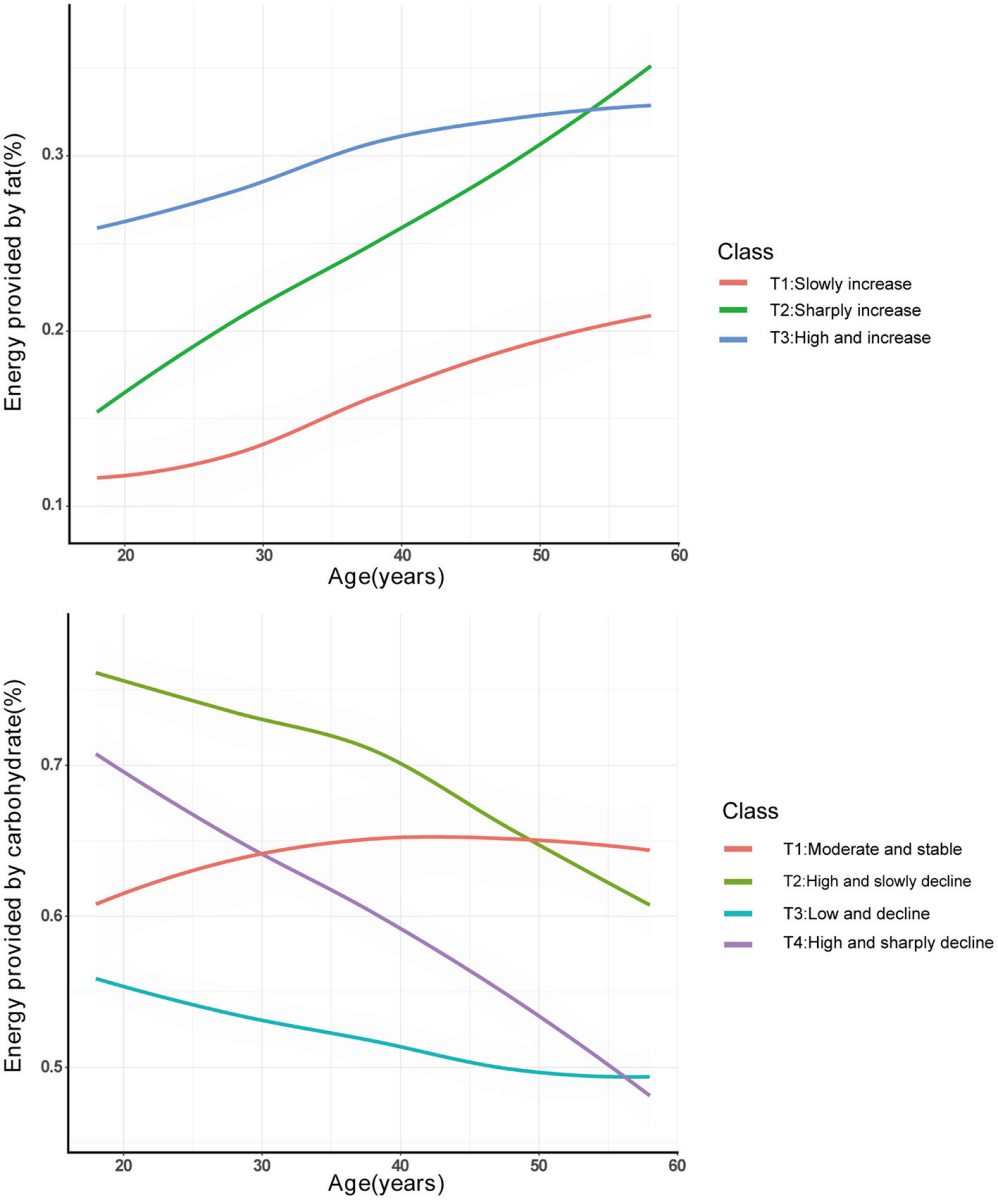
Trajectories of FER and CER from the CHNS (1991–2011) by latent class trajectory modeling. FER, energy provided by fat; CER, energy provided by carbohydrate.

Trajectories of FER and CER are shown in Figure 2. For FER, the first trajectory (T1: slow increase) reflects participants whose FER slowly increased from 10 to 20%. The second trajectory (T2: sharp increase) corresponds to participants whose FER sharply increased from 15 to 35%. The third trajectory (T3: high, and increase), corresponds to participants whose FER slowly increased from over 25% to over 30%. 20.9, 28.9, and 50.2% of the participants were in T1, T2, and T3, respectively. For CER, the first trajectory (T1: Moderate and stable) corresponds to participants with stable CER (55–65%). The second trajectory (T2: High and slow decline) corresponds to participants whose CER slowly decreased from over 75% to over 60%. The third trajectory (T3: Low and decline) corresponds to participants whose CER decreased from over 55–50%. The fourth trajectory (T4: High and sharp decline) corresponds to participants whose CER decreased from 70 to 45%. About 5.2, 21.6, 47.1, and 26.1% of participants were in T1, T2, T3, and T4, respectively.

### Characteristics Across Different Trajectory-Patterns

Seven patterns were identified by cross-grouping trajectories of FER and CER; trajectory-pattern 1 (FER: T1, CER: T1), 2 (FER: T1, CER: T2), 3 (FER: T2, CER: T2), 4 (FER: T2, CER: T3), 5 (FER: T2, CER: T4), 6 (FER: T3, CER: T3), 7 (FER: T3, CER: T4). The characteristics across different patterns were presented in Table 1. The baseline age ranged from 33.4 to 39.8 years. Average METs was not significantly different (*P* = 0.536), while other variables were significantly different across the patterns (*P* < 0.05).

**Table 1:** Characteristics of study variable across different trajectory-patterns^a^.

**Variables**	**Trajectory of FER T1**		**Trajectory of FER T2**			**Trajectory of FER T3**		***p*-value**
	**Trajectory of CER (T1)**	**Trajectory of CER (T2)**	**Trajectory of CER (T2)**	**Trajectory of CER (T3)**	**Trajectory of CER (T4)**	**Trajectory of CER (T3)**	**Trajectory of CER (T4)**	
	**Trajectory-pattern 1**	**Trajectory-pattern 2**	**Trajectory-pattern 3**	**Trajectory-pattern 4**	**Trajectory-pattern 5**	**Trajectory-pattern 6**	**Trajectory-pattern 7**	
Age (years)	39.8 ± 8.1	39.3 ± 9.6	37.0 ± 9.3	36.1 ± 10.4	32.4 ± 9.5	34.1 ± 11.0	33.4 ± 9.2	<0.001
Male [*n*, (%)]	219 (49.5)	843 (48.9)	201 (38.9)	165 (57.6)	1,091 (46.6)	2,126 (43.3)	199 (44.6)	<0.001
Mean BMI (kg/m^2^)	21.8 ± 2.8	22.2 ± 2.5	22.2 ± 2.6	22.7 ± 2.5	22.6 ± 2.5	22.4 ± 2.6	22.6 ± 2.6	<0.001
Mean PAL (MET-h/week)	321.2 ± 170.7	334.7 ± 167.5	356.9 ± 187.1	306.4 ± 188.2	340.8 ± 200.4	337.9 ± 225.5	335.1 ± 205.3	0.536
Mean energy intake(kJ/day)	9471.4 ± 1802.0	9758.0 ± 1789.5	9793.3 ± 1743.1	9599.7 ± 1809.1	9517.8 ± 1709.6	9331.8 ± 1745.9	9657.1 ± 1595.6	<0.001
Mean fat intake (g/day)	40.7 ± 11.9	47.3 ± 14.2	58.5 ± 16.0	67.8 ± 16.8	61.6 ± 18.2	82.3 ± 21.0	74.0 ± 16.4	<0.001
Mean carbohydrate intake (g/day)	408.1 ± 84.8	405.6 ± 86.3	388.2 ± 82.8	322.2 ± 67.1	358.1 ± 82.8	297.3 ± 65.2	341.1 ± 69.7	<0.001
Mean vegetable intake (g/day)	345.6 ± 125.0	347.2 ± 120.4	347.9 ± 115.8	315.9 ± 96.3	328.6 ± 104.2	312.3 ± 95.9	337.1 ± 101.8	<0.001
Mean Fruit intake (g/day)	15.7 ± 29.0	23.1 ± 42.8	28.1 ± 57.3	43.2 ± 63.4	35.4 ± 56.8	52.5 ± 70.0	42.6 ± 60.4	<0.001
Mean Whole-grain intake (g/day)	35.6 ± 63.1	27.5 ± 45.5	21.0 ± 36.8	14.4 ± 19.8	19.2 ± 36.4	14.7 ± 27.6	13.4 ± 25.9	<0.001
Mean Saturated fat intake (g/day)	8.1 ± 3.7	10.2 ± 4.2	11.0 ± 5.0	16.3 ± 6.2	13.7 ± 5.6	17.6 ± 7.7	13.6 ± 5.5	<0.001
Alcohol status [*n*, (%)]	152 (34.3)	550 (31.9)	120 (23.2)	156 (54.5)	691 (29.5)	1,564 (31.8)	121 (27.1)	<0.001
Smoking status [*n*, (%)]	163 (36.9)	563 (32.7)	136 (26.3)	129 (45.0)	646 (27.6)	1,270 (25.9)	129 (28.9)	<0.001
City medical insurance [*n*, (%)]	82 (18.5)	247 (14.3)	75 (14.5)	101 (35.3)	599 (25.6)	2,440 (49.7)	106 (23.8)	<0.001
Mean Urban index	42.6 ± 12.4	46.5 ± 13.5	49.6 ± 14.0	65.5 ± 15.7	55.3 ± 16.0	68.1 ± 15.7	56.3 ± 16.1	<0.001
Mean income (Yuan)	4891.4 ± 6861.5	4044.0 ± 4188.1	4730.3 ± 5754.1	9124.9 ± 11228.9	6267.5 ± 8972.1	9417.5 ± 11091.4	7576.8 ± 7767.4	<0.001

a *Continuous data were mean (SD). Generalized linear models adjusted for age and χ^2^-test were used to probe for differences in continuous variables and dichotomous variables. FER, energy provided by fat; CER, energy provided by carbohydrate; T, Trajectory; PAL, physical activity level*.

### Associations of Trajectory-Patterns With Cardio-Metabolic Disease and All-Cause Mortality

The relationship between trajectory-patterns and the risk of cardio-metabolic disease including obesity, diabetes, hypertension, and CVD and all-cause mortality, are presented in Table 2. Compared to the reference group, trajectory-pattern 2 (FER slowly increased from 10 to 20% and CER decreased from over 75% to over 60%) was significantly associated with an increased risk of diabetes [HR 1.42 [95% CI, 1.01–2.04]] and CVD [HR 1.59 [95% CI, 1.02–2.56]] with adjustment for demographic covariates, nutritional covariates, and BMI. However, these associations became non-significant after further adjustment for social environment covariates. The trajectory-pattern 4 (FER sharply increased from 15 to 35% and CER decreased from over 55 to 50%) was significantly associated with a higher risk of obesity [HR 1.83 [95% CI, 1.10–3.04]], diabetes [HR 1.80 [95% CI, 1.03–3.16]], and all-cause mortality [HR 2.29 [95% CI, 1.35–3.87]] with adjustment for full covariates. The trajectory-pattern 6 (FER slowly increased from over 25% to over 30% and CER decreased from over 55 to 50%) was significantly associated with a higher risk of obesity [HR 1.46 [95% CI, 1.02–2.17]], diabetes [HR 1.49 [95% CI, 1.01–2.25]], and all-cause mortality [HR 1.62 [95% CI, 1.18–2.22]] with adjustment for full covariates.

**Table 2:** Association of trajectory-patterns with risk of cardio-metabolic disease and all-cause mortality^a^.

**Trajectory-patterns**	**Case/*N***	**Model 1^b^**	**Model 2^c^**	**Model 3^d^**	**Model 4^e^**	**Model 5^f^**
		**Incidence of obesity**				
Trajectory-pattern 1	37/440	1 (reference)	1 (reference)	1 (reference)	–	1 (reference)
Trajectory-pattern 2	166/1,704	1.22 (0.85–1.74)	1.28 (0.89–1.84)	1.29 (0.89–1.85)	–	1.19 (0.82–1.71)
Trajectory-pattern 3	40/510	1.08 (0.69–1.69)	1.11 (0.70–1.76)	1.28 (0.80–2.03)	–	1.12 (0.70–1.78)
Trajectory-pattern 4	32/283	2.01 (1.25–3.23)	2.04 (1.25–3.33)	2.21 (1.34–3.66)	–	1.83 (1.10–3.04)
Trajectory-pattern 5	250/2,317	1.62 (1.14–2.28)	1.64 (1.14–2.38)	1.76 (1.20–2.56)	–	1.47 (1.01–2.16)
Trajectory-pattern 6	392/4,864	1.79 (1.27–2.51)	1.68 (1.16–2.42)	1.86 (1.26–2.73)	–	1.46 (1.02–2.17)
Trajectory-pattern 7	40/443	1.51 (0.96–2.37)	1.48 (0.93–2.37)	1.75 (1.09–2.81)	–	1.41 (0.87–2.27)
*P _*for trend*_*		<0.001	0.009	0.005	–	0.200
		**Incidence of diabetes**				
Trajectory-pattern 1	42/424	1 (reference)	1 (reference)	1 (reference)	1 (reference)	1 (reference)
Trajectory-pattern 2	141/1,627	0.92 (0.65–1.30)	1.44 (1.01–2.05)	1.47 (1.03–2.11)	1.42 (1.01–2.04)	1.41 (0.98–2.02)
Trajectory-pattern 3	32/483	0.79 (0.50–1.26)	1.37 (0.85–2.20)	1.56 (0.97–2.53)	1.39 (0.85–2.25)	1.35 (0.83–2.19)
Trajectory-pattern 4	22/257	1.39 (0.82–2.32)	2.01 (1.17–3.43)	2.04 (1.17–3.54)	1.82 (1.04–3.16)	1.80 (1.03–3.16)
Trajectory-pattern 5	130/2,125	0.78 (0.55–1.11)	1.49 (1.02–2.17)	1.51 (1.02–2.25)	1.36 (0.91–2.02)	1.31 (0.87–1.96)
Trajectory-pattern 6	197/4,366	0.98 (0.70–1.37)	1.56 (1.08–2.26)	1.69 (1.14–2.50)	1.54 (1.04–2.29)	1.49 (1.01–2.25)
Trajectory-pattern 7	22/410	0.85 (0.51–1.43)	1.51 (0.88–2.59)	1.66 (0.95–2.88)	1.48 (0.85–2.58)	1.41 (0.80–2.47)
*P _*for trend*_*		0.009	0.178	0.144	0.191	0.221
		**Incidence of hypertension**				
Trajectory-pattern 1	274/442	1 (reference)	1 (reference)	1 (reference)	1 (reference)	1 (reference)
Trajectory-pattern 2	780/1,719	0.67 (0.59–0.77)	1.06 (0.92–1.22)	1.09 (0.94–1.25)	1.05 (0.91–1.21)	0.99 (0.85–1.14)
Trajectory-pattern 3	191/516	0.57 (0.48–0.69)	1.05 (0.87–1.27)	1.14 (0.94–1.38)	1.00 (0.82–1.21)	0.92 (0.75–1.12)
Trajectory-pattern 4	93/286	0.63 (0.50–0.80)	1.01 (0.79–1.29)	1.10 (0.86–1.42)	0.99 (0.77–1.27)	0.89 (0.69–1.14)
Trajectory-pattern 5	754/2,327	0.53 (0.46–0.61)	1.12 (0.96–1.30)	1.21 (1.03–1.41)	1.10 (0.94–1.29)	0.97 (0.83–1.15)
Trajectory-pattern 6	1,186/4,893	0.56 (0.49–0.64)	1.10 (0.95–1.28)	1.22 (1.04–1.43)	1.13 (0.96–1.32)	0.98 (0.83–1.16)
Trajectory-pattern 7	179/445	0.62 (0.50–0.75)	1.28 (1.04–1.58)	1.43 (1.15–1.78)	1.30 (1.05–1.61)	1.11 (0.89–1.38)
*P _*for trend*_*		<0.001	0.419	0.046	0.198	0.753
		**Incidence of CVD**				
Trajectory-pattern 1	26/425	1 (reference)	1 (reference)	1 (reference)	1 (reference)	1 (reference)
Trajectory-pattern 2	71/1,631	0.74 (0.47–1.15)	1.62 (1.02–2.57)	1.66 (1.04–2.66)	1.59 (1.02–2.56)	1.48 (0.91–2.39)
Trajectory-pattern 3	13/485	0.51 (0.26–0.99)	1.47 (0.74–2.91)	1.63 (0.81–3.27)	1.34 (0.65–2.75)	1.22 (0.59–2.50)
Trajectory-pattern 4	6/257	0.58 (0.23–1.41)	1.42 (0.57–3.53)	1.44 (0.56–3.68)	1.29 (0.50–3.29)	1.13 (0.43–2.92)
Trajectory-pattern 5	46/2,126	0.43 (0.26–0.70)	1.59 (0.94–2.69)	1.60 (0.91–2.81)	1.44 (0.82–2.54)	1.22 (0.68–2.19)
Trajectory-pattern 6	63/4,371	0.48 (0.30–0.76)	1.40 (0.85–2.31)	1.49 (0.87–2.56)	1.35 (0.79–2.32)	1.14 (0.64–2.02)
Trajectory-pattern 7	10/411	0.41 (0.18–0.95)	1.47 (0.62–3.52)	1.59 (0.65–3.88)	1.43 (0.58–3.49)	1.22 (0.49–3.02)
*P _*for trend*_*		0.009	0.569	0.564	0.639	0.669
		**All-cause mortality**				
Trajectory-pattern 1	90/442	1 (reference)	1 (reference)	1 (reference)	1 (reference)	1 (reference)
Trajectory-pattern 2	182/1,724	0.54 (0.42–0.70)	1.20 (0.92–1.55)	1.28 (0.98–1.68)	1.30 (1.00–1.71)	1.23 (0.94–1.61)
Trajectory-pattern 3	41/517	0.46 (0.32–0.67)	1.50 (1.03–2.20)	1.60 (1.08–2.38)	1.63 (1.10–2.42)	1.48 (0.99–2.20)
Trajectory-pattern 4	20/286	0.55 (0.34–0.90)	2.10 (1.27–3.46)	2.47 (1.47–4.15)	2.70 (1.60–4.54)	2.29 (1.35–3.87)
Trajectory-pattern 5	93/2,341	0.25 (0.19–0.34)	1.29 (0.93–1.77)	1.51 (1.07–2.13)	1.60 (1.13–2.26)	1.39 (0.97–1.97)
Trajectory-pattern 6	168/4,911	0.36 (0.27–0.46)	1.66 (1.26–2.18)	1.86 (1.38–2.52)	1.94 (1.43–2.62)	1.62 (1.18–2.22)
Trajectory-pattern 7	15/446	0.25 (0.14–0.44)	1.39 (0.79–2.44)	1.55 (0.87–2.78)	1.62 (0.91–2.89)	1.41 (0.78–2.52)
*P _*for trend*_*		<0.001	0.006	0.002	0.001	0.049

a *Data was HR (95% CI). METs, individual income, intake of energy, fruit, vegetable, whole-grain, saturated fat, monounsaturated fat, and polyunsaturated fat. The urbanization index used mean values during the survey*.

b *Model 1 was a crude model*.

c *Model 2 was a further adjustment for demographic covariates, including age, sex, smoking status, alcohol status, mean METs (total metabolic equivalents), education levels and mean individual income*.

d *Model 3 was a further adjustment for nutritional covariates, including mean intake of energy, fruit, vegetables, whole-grains, saturated fatty acids, monounsaturated fatty acids, and polyunsaturated fatty acids*.

e *Model 4 was a further adjustment for mean BMI*.

f *Model 5 was a further adjustment for social environment covariates, including mean urbanization index, province and medical insurance*.

### Sensitivity Analysis

**Figure 3:**
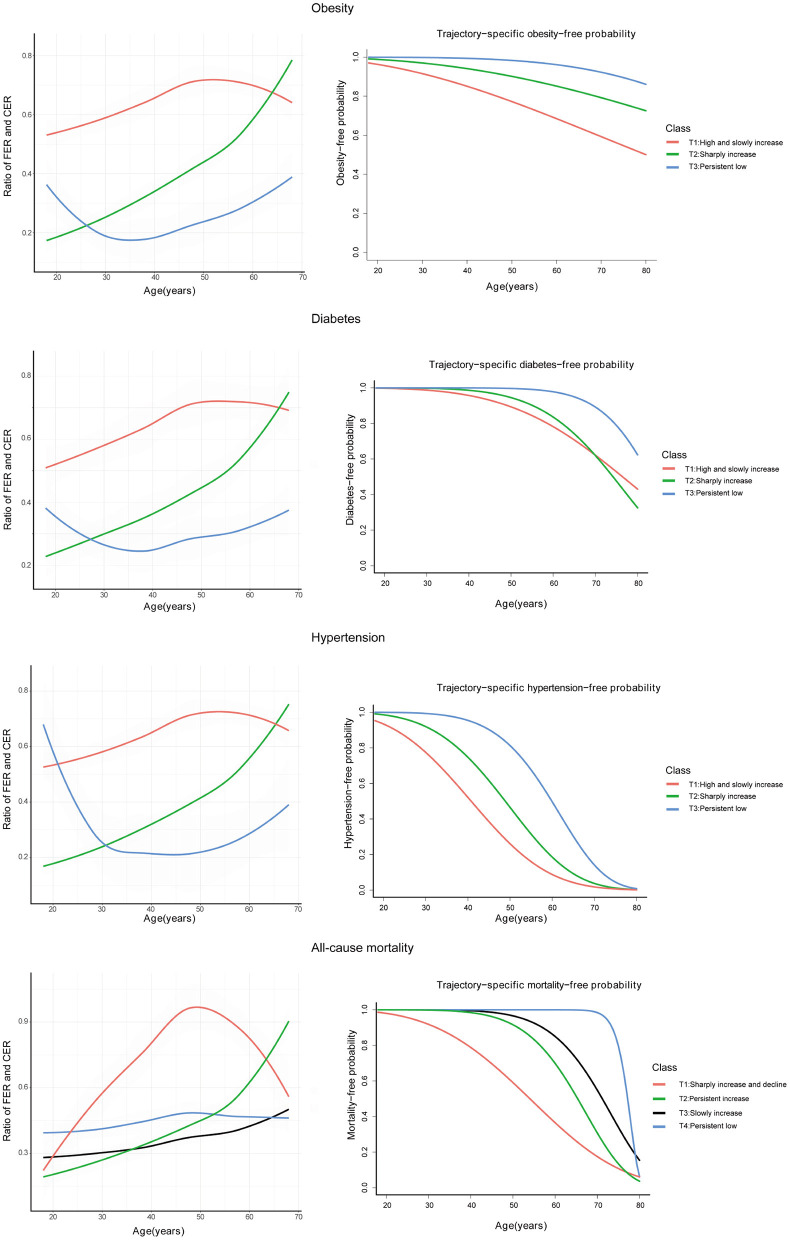
Trajectories of the ratio between FER and CER with time-to-outcomes by joint latent trajectory model and survival rate curve of these trajectories; FER, energy provided by fat; CER, energy provided by carbohydrate.

The trajectories of the ratio between FER and CER with time-to-outcomes and survival rate curves used to account for the ‘sick-quitter’ effect are presented in Figure 3. These results indicate that trajectories characterized by increased FER are in parallel with obesity-, diabetes-, hypertension-, and all-cause mortality-free probability, consistent with trajectory-patterns analysis above. Analyses of FER and CER measured using a single time point is presented in the Supplementary Table 2. The FER and CER measured in the 1991 survey were not significantly associated with all-cause mortality. Moreover, analyses of accumulated intake of FER and CER are presented in Supplementary Table 3. Compared to the lowest FER, the highest FER was significantly associated with a lower risk of all-cause mortality. In contrast, the highest CER was significantly associated with a higher risk of all-cause mortality compared to the lowest CER.

## Discussion

In this study, using longitudinal data gathered in China over 20 years, we observed that two trajectory-patterns of FER and CER were associated with the risk of obesity, diabetes, and all-cause mortality. Despite different dietary transition patterns over the course of adult life in the two trajectory-patterns, they can be consistently referred to as the high FER and low CER diet. Although low FER and high CER were associated with diabetes and CVD, these associations were probably influenced by the social environment, and low FER and high CER were not associated with all-cause mortality. These trajectory-patterns of FER and CER over adult life may provide important insights for nutritional policy and dietary guidelines.

The two trajectory-patterns mentioned above show FER sharply increasing from 15 to 35%, or slowly increasing from over 25% to over 30%, and the CER (in both cases) decreasing from over 55 to 50%. This indicates that, regardless of how fast FER increased over the adult life, a high FER and low CER diet could increase the risk of obesity, diabetes, and all-cause mortality.

This study also examined the relationships between single baseline measures or cumulative intake of FER and CER and these outcomes, which were frequently used in previous studies (4, 5, 15–17). Results indicate that associations of a single baseline measure of FER and CER with all-cause mortality were non-significant, while the associations of cumulative intake of FER and CER with all-cause mortality showed a completely different picture from the trajectory-pattern analysis. Taken together, these results indicate that a single baseline measure or average intake mask the variation intake over time, probably leading to a misestimation of the health impacts of FER and CER. In addition, the trajectory-pattern is closer to reflecting real-world intakes, which could more accurately capture health impacts. High fat-induced obesity/diabetes has been extensively studied in animals (26–30). Some ecological studies have indicated that the time trend of dietary fat increase parallels the increase in the prevalence of obesity and diabetes (31–34), which could partially support the associations in this study. Some nutritionists disagree with these observations because of limitations such as lack of adjustment for confounding factors (35, 36), the use of trajectory-pattern analysis may solve these issues.

Besides the two trajectory-patterns above, this study also observed that a trajectory-pattern was associated with a higher risk of CVD and diabetes. This trajectory-pattern was characterized by FER slowly increasing from 10 to 20%, and CER of over 70% during early and middle adulthood, decreasing to over 60% during late adulthood. This can be referred to as a persistently low FER and high CER diet. Similarly, previous studies have suggested that a high carbohydrate diet is associated with higher blood glucose levels and risk of CVD (37, 38). However, this study found that these associations were not significant after adjustment for social environment covariates, such as urbanization and medical insurance, indicating that the social environment may influence the relationship between a low FER/high CER diet and the risk of diabetes and CVD in this population.

Although this study had a smaller sample size than previous large observational studies or randomized controlled trials (RCTs), it has some unique strengths and implications. Large, long-term RCTs have been regarded as the only solution for settling this debate (39). However, the uniqueness of nutrition limits the crucial characteristics of RCTs, such as high compliance and low drop-out rate in large, long-term RCTs (40). For instance, most of the participants randomly assigned to the low-fat group cannot achieve a fat reduction target of 20% after more than 8 years of follow-up (41). With rapid economic growth, a dietary transition from high CER to high FER is occurring in the Chinese population. This transition provides a unique quasi-experimental model to compare health impacts between participants who changed their diet to high FER and who persistently stay in a low FER diet. This quasi-experiment model may provide insights into the health impacts of high FER and low CER diets with adjustment for multiple confounding variables. Previous large observational studies have frequently examined the relationship between macronutrients measured at a single time point and outcomes during long-term follow-up, ignoring the variation in macronutrient intake. In contrast, our study may be closer and more accurate in reflecting real-world intake. Moreover, due to nutritional trade-off effects, this study did not analyze the trajectories of FER and CER separately or use a dietary replacement model, instead cross-grouping these trajectories to capture real-world dietary trajectory-patterns.

This study does, however, have some limitations. First, it included only Asian participants, limiting its generalizability to other ethnic populations. Compared with other ethnicities, absorption and metabolic rates of carbohydrates tend to be higher, whereas that of fat tend to be lower in Asian people (42, 43). Therefore, the adverse effect may occur during the transition to high FER and low CER diet in the Asian population. Second, as in any observational study, our study had the possibility of residual confounding factor and measurement error. In mitigation, however, we accounted for various confounding factors not commonly considered in previous studies, even including medical insurance and urbanization. Moreover, dietary assessment in the CHNS was based on a combination of three consecutive days of detailed food consumption information with a weighing technique. Although this method is more accurate than the food frequency questionnaire, it cannot reflect long-term dietary habits (44). The changing trends of dietary transition reflected by this method, however, are similar to those of the food frequency questionnaire. In addition, the alcohol consumption level in the questionnaire was based on consumption frequency rather than daily alcohol intake, which cannot accurately collect the subjects' alcohol intake levels. To a certain extent, though, consumption frequency can reflect the subjects' long-term alcohol consumption habits. Therefore, residual confounding and measurement errors may slightly influence the study results. Third, CVD and diabetes were based on self-reported information, and only blood samples were used to diagnose diabetes in the 2009 survey. The incidence of CVD and diabetes was therefore relatively low throughout the CHNS, which may influence the relationship between trajectory-patterns and outcomes. Finally, our study has only shown the trajectories of total fat and carbohydrate intake in adulthood and their impacts on mortality. Further studies are needed to assess the relationship between types of fat and carbohydrate and mortality.

## Conclusions

This study demonstrated that changing to a high FER and low CER diet was associated with obesity, diabetes, and all-cause mortality. These trajectory-patterns of FER and CER over the adult life-course may provide insights into nutritional policy and dietary guidelines.

## Data Availability Statement

Data in this study can be downloaded from http://www.cpc.unc.edu/projects/china.

## Ethics Statement

The studies involving human participants were reviewed and approved by Institutional Review Committees of the University of North Carolina at Chapel Hill, NC, USA, and the China National Institute of Nutrition and Food Safety at the Chinese Center for Disease Control and Prevention, Beijing, China. The patients/participants provided their written informed consent to participate in this study.

## Author Contributions

CS, TH, and YL: concept. JG, XG, and TH: methodology. XG, XX, and WW: software. WH and XW: validation. JG and XX: original draft preparation. XG and TH: review and editing. WH and JG: visualization. CS and YL: supervision. CS and TH: funding acquisition. All authors have read and agreed to the published version of the manuscript.

## Conflict of Interest

The authors declare that the research was conducted in the absence of any commercial or financial relationships that could be construed as a potential conflict of interest.

## Publisher's Note

All claims expressed in this article are solely those of the authors and do not necessarily represent those of their affiliated organizations, or those of the publisher, the editors and the reviewers. Any product that may be evaluated in this article, or claim that may be made by its manufacturer, is not guaranteed or endorsed by the publisher.

## Abbreviations

CER: Carbohydrate-to-energy ratio
CHNS: The China Health and Nutrition Survey
CI: Confidence interval
CVD: Cardiovascular disease
FER: Fat-to-energy ratio
HR: Hazard ratio
LCTM: Latent class trajectory modeling
METs: Metabolic equivalents
*P*: Probability
RCT: Randomized controlled trial
SD: Standard deviation.
