# Supplementary material for: Twenty-Year Trajectory-Patterns of Percentage Energy From Dietary Fat vs. Carbohydrate Throughout Adult Life and Associations With Cardio-Metabolic Disease and All-Cause Mortality
Source: Front Nutr. 2021 Sep 6;8:701188. doi: 10.3389/fnut.2021.701188 (PMC8450393; doi:10.3389/fnut.2021.701188)
Supplement: Supplementary file 1 [file Table_1.docx]

***Supplementary Material***

**1 Supplementary Table 1** Characteristic of the study population from the China Health and Nutrition

|  |  | Survey years | | | | | | |
| --- | --- | --- | --- | --- | --- | --- | --- | --- |
| Variables | 1991  (N=5931) | 1993  (N=6042) | 1997  (N=6196) | 2000  (N=6973) | 2004  (N=6542) | 2006  (N=6508) | 2009  (N=6534) | 2011  (N=5967) |
| Age (years) | 35.8(11.1) | 37.6(11.2) | 40.6(11.3) | 42.8(11.3) | 46.2(11.6) | 48.0(11.5) | 49.8(12.0) | 51.8(12.0) |
| Male [(n, (%)) | 2944(49.6) | 2936(48.6) | 2994(48.3) | 3285(47.1) | 3000(45.9) | 2940(45.2) | 2929(44.8) | 2636(44.2) |
| BMI (kg/m^2^) | 21.3(2.3) | 21.6(2.4) | 22.0(2.6) | 22.6(2.8) | 22.7(2.9) | 22.9(3.2) | 23.1(3.1) | 23.5(4.5) |
| METs (h /week) | 375.4(281.7) | 475.3(566.7) | 328.9(420.1) | 340.0(456.7) | 206.2(188.4) | 217.2(195.4) | 290.1(229.8) | 281.8(215.5) |
| High school education [(n, (%)) | 277(4.7) | 277(4.6) | 337(5.4) | 567(8.1) | 592(9.0) | 724(11.1) | 739(11.3) | 681(11.4) |
| Energy intake (kJ/day) | 10929.3(2435.3) | 10390.5(2446.6) | 9786.7(2451.3) | 9526.5(2509.8) | 9246.7(2554.1) | 9135.4(2631.3) | 8932.9(2558.2) | 8361.5(2584.9) |
| fat intake (g/day) | 61.4(34.5) | 61.4(32.4) | 64.1(34.1) | 70.9(37.1) | 68.6(36.8) | 70.7(36.6) | 73.3(34.7) | 71.0(33.8) |
| carbohydrate intake (g/day) | 433.5(122.5) | 403.5(122.2) | 366.9(118.5) | 337.5(103.1) | 325.2(102.7) | 314.3(109.5) | 297.3(98.9) | 270.6(102.1) |
| Vegetable intake (g/day) | - | - | 318.5(162.4) | 333.1(192.7) | 349.1(202.6) | 337.6(185.2) | 328.5(169.9) | 309.3(166.6) |
| Fruit intake (g/day) | - | - | 17.4(95.1) | 19.1(82.0) | 22.8(70.2) | 51.1(151.7) | 53.6(108.8) | 77.8(131.5) |
| Whole-grain intake (g/day) | - | - | 22.0(63.4) | 19.1(54.6) | 17.4(56.0) | 13.0(39.8) | 16.6(50.8) | 21.4(60.0) |
| Saturated fat intake (g/day) | - | - | 11.3(7.7) | 12.8(9.9) | 13.8(11.2) | 15.3(11.4) | 16.2(10.9) | 15.8(10.4) |
| Alcohol consumption [(n, (%)) | - | 1914(31.7) | 2109(34.0) | 2157(30.9) | 2152(32.9) | 2082(32.0) | 2143(32.8) | 1905(31.9) |
| Smoking [(n, (%)) | 1816(30.6) | 1841(30.5) | 2059(33.2) | 1961(28.1) | 1945(29.7) | 1802(27.7) | 1838(28.1) | 1602(26.8) |
| Living in city [(n, (%)) | 1845(31.1) | 1864(30.9) | 1910(30.8) | 2183(31.3) | 2050(31.3) | 2048(31.5) | 2012(30.8) | 1745(29.2) |
| Urbanicity index | 45.7(15.9) | 47.6(16.1) | 51.6(17.8) | 57.8(18.1) | 60.8(20.0) | 63.0(20.1) | 65.8(19.1) | 66.3(18.5) |
| Individual’s income(Yuan) | 1412.7(1418.3) | 2076.5(2521.6) | 4201.5(4436.0) | 5232.7(6475.5) | 6689.3(8674.2) | 9216.9(13242.9) | 15815.5(24739.5) | 19814.0(26602.6) |

METs, total metabolic equivalents

**2 Supplementary Table 2** Association of dietary FER or CER measured at the 1991survey and cardio-metabolic disease and all-cause mortality ^a^

|  |  | **Incidence of obesity** | | | |  |
| --- | --- | --- | --- | --- | --- | --- |
|  | Case/N | Model 1^b^ | Model 2^c^ | Model 3^d^ | Model 4^e^ | Model 5^f^ |
|  |  | HR (95% CI) | HR (95% CI) | HR (95% CI) | HR (95% CI) | HR (95% CI) |
| FER ^g^ |  |  |  |  |  |  |
| Q1 | 92/1068 | 1 (Ref.) | 1 (Ref.) | 1 (Ref.) | - | 1 (Ref.) |
| Q2 | 108/1069 | 1.27(0.96-1.68) | 1.21(0.92-1.61) | 1.28(0.96-1.69) | - | 1.16(0.87-1.54) |
| Q3 | 109/1069 | 1.39(1.05-1.83) | 1.28(0.96-1.70) | 1.37(1.03-1.84) | - | 1.24(0.92-1.66) |
| Q4 | 111/1068 | 1.49(1.13-1.96) | 1.34(1.00-1.78) | 1.41(1.05-1.89) | - | 1.20(0.88-1.62) |
| Q5 | 91/1069 | 1.22(0.91-1.63) | 1.04(0.77-1.41) | 1.06(0.77-1.44) | - | 0.83(0.60-1.16) |
| *p* for trend |  | 0.058 | 0.170 | 0.055 | - | 0.044 |
| CER^7^ |  |  |  |  |  |  |
| Q1 | 104/1068 | 1 (Ref.) | 1 (Ref.) | 1 (Ref.) | - | 1 (Ref.) |
| Q2 | 101/1070 | 0.94(0.71-1.23) | 0.95(0.72-1.26) | 0.99(0.75-1.30) | - | 1.04(0.79-1.38) |
| Q3 | 110/1068 | 0.98(0.75-1.28) | 1.02(0.78-1.34) | 1.07(0.81-1.41) | - | 1.19(0.90-1.58) |
| Q4 | 108/1068 | 0.87(0.66-1.14) | 0.95(0.72-1.26) | 0.98(0.73-1.31) | - | 1.11(0.82-1.50) |
| Q5 | 88/1069 | 0.66(0.49-0.87) | 0.74(0.55-1.00) | 0.73(0.54-1.00) | - | 0.91(0.65-1.26) |
| *p* for trend |  | 0.031 | 0.233 | 0.141 | - | 0.393 |
|  |  |  |  |  |  |  |
|  |  | **Incidence of diabetes** | | | |  |
| FER |  |  |  |  |  |  |
| Q1 | 59/961 | 1 (Ref.) | 1 (Ref.) | 1 (Ref.) | 1 (Ref.) | 1 (Ref.) |
| Q2 | 87/916 | 1.62(1.16-2.25) | 1.56(1.12-2.17) | 1.64(1.17-2.30) | 1.62(1.15-2.26) | 1.55(1.10-2.18) |
| Q3 | 70/878 | 1.44(1.01-2.03) | 1.27(0.89-1.80) | 1.36(0.95-1.96) | 1.31(0.91-1.89) | 1.27(0.88-1.83) |
| Q4 | 70/847 | 1.50(1.06-2.12) | 1.31(0.92-1.88) | 1.39(0.96-2.00) | 1.29(0.89-1.86) | 1.22(0.83-1.78) |
| Q5 | 60/848 | 1.33(0.93-1.90) | 1.10(0.75-1.59) | 1.14(0.77-1.68) | 1.10(0.74-1.62) | 1.02(0.68-1.54) |
| *p* for trend |  | 0.059 | 0.078 | 0.040 | 0.047 | 0.060 |
| CER |  |  |  |  |  |  |
| Q1 | 68/839 | 1 (Ref.) | 1 (Ref.) | 1 (Ref.) | 1 (Ref.) | 1 (Ref.) |
| Q2 | 68/851 | 0.94(0.67-1.32) | 1.02(0.73-1.43) | 1.05(0.75-1.47) | 1.05(0.75-1.48) | 1.07(0.76-1.51) |
| Q3 | 58/878 | 0.76(0.53-1.08) | 0.85(0.60-1.22) | 0.87(0.61-1.25) | 0.90(0.62-1.29) | 0.92(0.64-1.33) |
| Q4 | 88/918 | 1.03(0.75-1.42) | 1.22(0.88-1.69) | 1.25(0.89-1.75) | 1.30(0.93-1.82) | 1.33(0.94-1.90) |
| Q5 | 64/964 | 0.69(0.49-0.97) | 0.84(0.59-1.21) | 0.83(0.57-1.21) | 0.89(0.61-1.30) | 0.95(0.63-1.41) |
| *p* for trend |  | 0.071 | 0.149 | 0.106 | 0.120 | 0.159 |
|  |  | **Incidence of hypertension** | | | |  |
| FER |  |  |  |  |  |  |
| Q1 | 475/1070 | 1 (Ref.) | 1 (Ref.) | 1 (Ref.) | 1 (Ref.) | 1 (Ref.) |
| Q2 | 446/1071 | 1.00(0.88-1.14) | 1.02(0.90-1.16) | 1.06(0.93-1.21) | 1.07(0.93-1.21) | 1.03(0.90-1.18) |
| Q3 | 431/1071 | 1.03(0.91-1.18) | 1.01(0.88-1.15) | 1.07(0.93-1.23) | 1.05(0.91-1.20) | 1.02(0.89-1.18) |
| Q4 | 394/1069 | 0.97(0.85-1.11) | 0.97(0.84-1.11) | 1.02(0.88-1.17) | 0.98(0.85-1.13) | 0.94(0.81-1.09) |
| Q5 | 367/1070 | 0.89(0.78-1.02) | 0.88(0.77-1.02) | 0.92(0.80-1.07) | 0.90(0.78-1.05) | 0.86(0.74-1.01) |
| *p* for trend |  | 0.283 | 0.310 | 0.273 | 0.200 | 0.131 |
| CER |  |  |  |  |  |  |
| Q1 | 371/1070 | 1 (Ref.) | 1 (Ref.) | 1 (Ref.) | 1 (Ref.) | 1 (Ref.) |
| Q2 | 404/1070 | 1.08(0.94-1.24) | 1.13(0.98-1.30) | 1.14(0.99-1.32) | 1.15(1.00-1.33) | 1.14(0.99-1.32) |
| Q3 | 419/1070 | 1.07(0.93-1.24) | 1.13(0.98-1.31) | 1.14(0.99-1.32) | 1.17(1.01-1.35) | 1.18(1.02-1.36) |
| Q4 | 440/1071 | 1.05(0.91-1.20) | 1.09(0.95-1.26) | 1.09(0.94-1.26) | 1.13(0.97-1.30) | 1.13(0.97-1.31) |
| Q5 | 479/1070 | 1.08(0.94-1.24) | 1.10(0.95-1.27) | 1.06(0.91-1.23) | 1.10(0.95-1.28) | 1.13(0.97-1.33) |
| *p* for trend |  | 0.769 | 0.400 | 0.278 | 0.217 | 0.214 |
|  | **Incidence of CVD** | | | | |  |
| FER |  |  |  |  |  |  |
| Q1 | 38/963 | 1 (Ref.) | 1 (Ref.) | 1 (Ref.) | 1 (Ref.) | 1 (Ref.) |
| Q2 | 32/917 | 0.90(0.56-1.45) | 0.86(0.53-1.38) | 0.88(0.54-1.42) | 0.87(0.54-1.41) | 0.83(0.51-1.35) |
| Q3 | 28/878 | 0.87(0.53-1.42) | 0.73(0.44-1.20) | 0.78(0.47-1.31) | 0.77(0.46-1.29) | 0.71(0.42-1.20) |
| Q4 | 30/847 | 0.97(0.60-1.57) | 0.84(0.51-1.39) | 0.92(0.55-1.54) | 0.87(0.52-1.46) | 0.76(0.44-1.30) |
| Q5 | 18/852 | 0.59(0.34-1.04) | 0.46(0.26-0.83) | 0.51(0.28-0.94) | 0.49(0.27-0.90) | 0.40(0.21-0.76) |
| *p* for trend |  | 0.458 | 0.128 | 0.250 | 0.220 | 0.081 |
| CER |  |  |  |  |  |  |
| Q1 | 18/842 | 1 (Ref.) | 1 (Ref.) | 1 (Ref.) | 1 (Ref.) | 1 (Ref.) |
| Q2 | 31/852 | 1.64(0.92-2.94) | 1.88(1.05-3.38) | 1.85(1.03-3.33) | 1.86(1.04-3.35) | 1.99(1.10-3.59) |
| Q3 | 25/878 | 1.26(0.68-2.31) | 1.53(0.83-2.82) | 1.46(0.79-2.71) | 1.53(0.82-2.84) | 1.72(0.91-3.22) |
| Q4 | 33/919 | 1.50(0.84-2.66) | 1.90(1.05-3.42) | 1.76(0.96-3.20) | 1.84(1.01-3.36) | 2.23(1.19-4.16) |
| Q5 | 39/966 | 1.65(0.94-2.89) | 2.23(1.24-4.00) | 2.02(1.10-3.70) | 2.14(1.17-3.93) | 2.75(1.44-5.25) |
| *p* for trend |  | 0.380 | 0.085 | 0.173 | 0.135 | 0.036 |
|  | **All-cause mortality** | | | | |  |
| FER |  |  |  |  |  |  |
| Q1 | 87/1070 | 1 (Ref.) | 1 (Ref.) | 1 (Ref.) | 1 (Ref.) | 1 (Ref.) |
| Q2 | 75/1071 | 0.92(0.67-1.25) | 0.97(0.71-1.33) | 0.95(0.69-1.30) | 0.95(0.69-1.29) | 0.99(0.72-1.35) |
| Q3 | 70/1071 | 0.93(0.68-1.28) | 1.01(0.74-1.40) | 0.95(0.68-1.33) | 0.96(0.69-1.33) | 0.99(0.71-1.39) |
| Q4 | 52/1069 | 0.73(0.51-1.03) | 0.87(0.61-1.24) | 0.85(0.59-1.21) | 0.86(0.60-1.23) | 0.93(0.64-1.34) |
| Q5 | 55/1070 | 0.78(0.56-1.10) | 0.94(0.66-1.34) | 0.92(0.64-1.32) | 0.93(0.65-1.34) | 1.01(0.69-1.48) |
| *p* for trend |  | 0.368 | 0.938 | 0.936 | 0.953 | 0.992 |
| CER |  |  |  |  |  |  |
| Q1 | 67/1070 | 1 (Ref.) | 1 (Ref.) | 1 (Ref.) | 1 (Ref.) | 1 (Ref.) |
| Q2 | 46/1070 | 0.66(0.45-0.96) | 0.70(0.48-1.03) | 0.69(0.47-1.01) | 0.68(0.47-1.00) | 0.68(0.47-1.00) |
| Q3 | 63/1070 | 0.86(0.61-1.22) | 0.86(0.61-1.22) | 0.82(0.57-1.17) | 0.81(0.57-1.16) | 0.78(0.54-1.11) |
| Q4 | 81/1071 | 1.00(0.72-1.39) | 0.94(0.67-1.31) | 0.92(0.65-1.29) | 0.90(0.64-1.27) | 0.86(0.60-1.23) |
| Q5 | 82/1070 | 0.95(0.69-1.32) | 0.82(0.58-1.15) | 0.82(0.57-1.17) | 0.81(0.57-1.15) | 0.74(0.51-1.07) |
| *p* for trend |  | 0.173 | 0.408 | 0.375 | 0.361 | 0.296 |

^a^ Data was HR (95%CI). METs, individual’s income, intake of energy, fruit, vegetable, whole-grain, saturated fat, monounsaturated fat and polyunsaturated fat, urbanization index were used mean values during the survey.

^b^ Model 1 was crude model.

^c^ Model 2 was further adjustment for demographic covariates including age, sex, smoking status, alcohol status, mean METs (total metabolic equivalents), education levels, mean individual’s income.

^d^ Model 3 was further adjustment for nutritional covariates including mean intake of energy, fruit, vegetable, whole-grain, saturated fatty acid, monounsaturated fatty acid and polyunsaturated fatty acid.

^e^ Model 4 was further adjustment for mean BMI.

^f^ Model 5 was further adjustment for social environment covariates including mean urbanization index, province, medical insurance.

^g^ FER, energy provided by dietary fat; CER, energy provided by dietary carbohydrate.

**3 Supplementary Table 3** Association of accumulation dietary FER or CER with cardio-metabolic disease and all-cause mortality ^a^

| Accumulation of FER and CER | Case/N | **Incidence of obesity** | | | |  |
| --- | --- | --- | --- | --- | --- | --- |
|  |  | Model 1^b^ | Model 2^c^ | Model 3^d^ | Model 4^e^ | Model 5^f^ |
|  |  | HR (95% CI) | HR (95% CI) | HR (95% CI) | HR (95% CI) | HR (95% CI) |
| FER ^g^ |  |  |  |  |  |  |
| Q1 | 160/2142 | 1 (Ref.) | 1 (Ref.) | 1 (Ref.) | 1 (Ref.) | 1 (Ref.) |
| Q2 | 183/2166 | 1.07(0.86-1.32) | 1.02(0.82-1.26) | 1.13(0.91-1.40) |  | 1.06(0.85-1.32) |
| Q3 | 210/2171 | 1.29(1.05-1.59) | 1.18(0.95-1.46) | 1.31(1.05-1.64) |  | 1.17(0.93-1.46) |
| Q4 | 237/2172 | 1.55(1.27-1.90) | 1.36(1.10-1.68) | 1.57(1.24-1.98) |  | 1.33(1.05-1.69) |
| Q5 | 187/2168 | 1.50(1.22-1.86) | 1.23(0.97-1.55) | 1.35(1.04-1.75) |  | 1.07(0.82-1.41) |
| *p* for trend |  | <0.001 | 0.021 | 0.002 |  | 0.062 |
| CER^7^ |  |  |  |  |  |  |
| Q1 | 188/2166 | 1 (Ref.) | 1 (Ref.) | 1.00(ref.) |  | 1.00(ref.) |
| Q2 | 221/2175 | 0.96(0.79-1.16) | 1.02(0.84-1.24) | 1.09(0.89-1.33) |  | 1.16(0.95-1.43) |
| Q3 | 221/2173 | 0.90(0.74-1.10) | 1.00(0.82-1.23) | 1.07(0.86-1.32) |  | 1.20(0.96-1.50) |
| Q4 | 184/2162 | 0.70(0.57-0.86) | 0.81(0.65-1.01) | 0.86(0.67-1.09) |  | 1.05(0.82-1.34) |
| Q5 | 163/2143 | 0.66(0.54-0.82) | 0.80(0.63-1.02) | 0.79(0.60-1.04) |  | 1.04(0.78-1.39) |
| *p* for trend |  | <0.001 | 0.057 | 0.033 |  | 0.289 |
|  |  |  |  |  |  |  |
|  |  | **Incidence of diabetes** | | | |  |
| FER |  |  |  |  |  |  |
| Q1 | 98/1884 | 1 (Ref.) | 1 (Ref.) | 1 (Ref.) | 1 (Ref.) | 1 (Ref.) |
| Q2 | 136/1968 | 1.31(1.01-1.70) | 1.29(0.99-1.68) | 1.40(1.07-1.83) | 1.37(1.04-1.79) | 1.36(1.04-1.79) |
| Q3 | 131/2012 | 1.34(1.03-1.74) | 1.16(0.88-1.52) | 1.24(0.93-1.65) | 1.16(0.87-1.54) | 1.13(0.85-1.52) |
| Q4 | 120/2026 | 1.35(1.04-1.77) | 1.12(0.85-1.49) | 1.24(0.91-1.68) | 1.12(0.82-1.53) | 1.09(0.79-1.49) |
| Q5 | 127/2053 | 1.95(1.50-2.55) | 1.43(1.07-1.91) | 1.52(1.09-2.12) | 1.38(0.99-1.92) | 1.30(0.92-1.84) |
| *p* for trend |  | <0.001 | 0.095 | 0.064 | 0.097 | 0.120 |
| CER |  |  |  |  |  |  |
| Q1 | 131/2055 | 1 (Ref.) | 1 (Ref.) | 1.00(ref.) | 1 (Ref.) | 1 (Ref.) |
| Q2 | 121/2020 | 0.67(0.53-0.86) | 0.76(0.59-0.98) | 0.81(0.62-1.05) | 0.78(0.60-1.01) | 0.79(0.61-1.03) |
| Q3 | 131/1998 | 0.67(0.53-0.86) | 0.82(0.63-1.06) | 0.87(0.66-1.14) | 0.87(0.66-1.15) | 0.90(0.68-1.19) |
| Q4 | 132/1981 | 0.62(0.49-0.80) | 0.84(0.64-1.09) | 0.90(0.67-1.20) | 0.93(0.69-1.25) | 0.98(0.72-1.33) |
| Q5 | 97/1893 | 0.48(0.37-0.63) | 0.65(0.48-0.87) | 0.66(0.46-0.93) | 0.70(0.50-0.99) | 0.74(0.52-1.07) |
| *p* for trend |  | <0.001 | 0.056 | 0.110 | 0.126 | 0.168 |
|  |  | **Incidence of hypertension** | | | |  |
| FER |  |  |  |  |  |  |
| Q1 | 731/2166 | 1 (Ref.) | 1 (Ref.) | 1.00(ref.) | 1 (Ref.) | 1 (Ref.) |
| Q2 | 724/2179 | 0.91(0.83-1.01) | 0.93(0.84-1.03) | 0.99(0.89-1.11) | 0.99(0.89-1.10) | 0.95(0.86-1.06) |
| Q3 | 748/2179 | 1.00(0.91-1.11) | 0.95(0.86-1.06) | 1.04(0.93-1.17) | 1.01(0.90-1.13) | 0.93(0.83-1.04) |
| Q4 | 731/2179 | 1.05(0.95-1.17) | 1.03(0.93-1.15) | 1.16(1.03-1.31) | 1.08(0.96-1.22) | 0.98(0.87-1.11) |
| Q5 | 609/2183 | 1.06(0.95-1.18) | 1.00(0.89-1.13) | 1.11(0.97-1.27) | 1.03(0.90-1.18) | 0.90(0.78-1.04) |
| *p* for trend |  | 0.048 | 0.300 | 0.062 | 0.547 | 0.508 |
| CER |  |  |  |  |  |  |
| Q1 | 636/2183 | 1 (Ref.) | 1 (Ref.) | 1.00(ref.) | 1 (Ref.) | 1 (Ref.) |
| Q2 | 731/2180 | 0.93(0.84-1.04) | 0.96(0.87-1.08) | 0.97(0.87-1.09) | 0.94(0.84-1.05) | 0.98(0.87-1.09) |
| Q3 | 722/2181 | 0.86(0.77-0.95) | 0.91(0.81-1.01) | 0.90(0.80-1.01) | 0.91(0.81-1.02) | 0.95(0.84-1.07) |
| Q4 | 739/2177 | 0.82(0.74-0.91) | 0.89(0.80-1.00) | 0.86(0.76-0.97) | 0.89(0.79-1.01) | 0.99(0.87-1.13) |
| Q5 | 715/2165 | 0.85(0.77-0.95) | 0.91(0.81-1.03) | 0.82(0.72-0.95) | 0.86(0.75-1.00) | 1.01(0.87-1.17) |
| *p* for trend |  | 0.002 | 0.320 | 0.038 | 0.347 | 0.869 |
|  | **Incidence of CVD** | | | | |  |
| FER |  |  |  |  |  |  |
| Q1 | 50/1897 | 1 (Ref.) | 1 (Ref.) | 1.00(ref.) | 1 (Ref.) | 1 (Ref.) |
| Q2 | 43/1970 | 0.79(0.52-1.19) | 0.84(0.56-1.27) | 0.89(0.58-1.35) | 0.87(0.57-1.33) | 0.84(0.55-1.29) |
| Q3 | 55/2007 | 1.08(0.73-1.58) | 1.02(0.69-1.52) | 1.06(0.70-1.62) | 0.99(0.65-1.51) | 0.90(0.58-1.40) |
| Q4 | 42/2027 | 0.90(0.60-1.36) | 0.88(0.57-1.35) | 0.94(0.58-1.51) | 0.83(0.52-1.34) | 0.72(0.43-1.18) |
| Q5 | 50/2056 | 1.43(0.96-2.12) | 1.31(0.85-2.02) | 1.39(0.84-2.29) | 1.23(0.74-2.04) | 0.97(0.57-1.66) |
| *p* for trend |  | 0.055 | 0.268 | 0.327 | 0.439 | 0.567 |
| CER |  |  |  |  |  |  |
| Q1 | 39/2057 | 1 (Ref.) | 1 (Ref.) | 1.00(ref.) | 1 (Ref.) | 1 (Ref.) |
| Q2 | 59/2022 | 1.14(0.76-1.71) | 1.21(0.80-1.83) | 1.28(0.83-1.96) | 1.23(0.80-1.89) | 1.34(0.87-2.07) |
| Q3 | 42/2000 | 0.75(0.48-1.16) | 0.83(0.52-1.31) | 0.87(0.53-1.41) | 0.87(0.53-1.41) | 0.98(0.60-1.60) |
| Q4 | 53/1986 | 0.87(0.57-1.32) | 1.03(0.66-1.62) | 1.12(0.68-1.84) | 1.18(0.72-1.95) | 1.48(0.88-2.48) |
| Q5 | 47/1892 | 0.83(0.54-1.28) | 0.93(0.58-1.51) | 0.99(0.57-1.74) | 1.08(0.61-1.88) | 1.42(0.78-2.58) |
| *p* for trend |  | 0.253 | 0.434 | 0.386 | 0.456 | 0.245 |
|  | **All-cause mortality** | | | | |  |
| FER |  |  |  |  |  |  |
| Q1 | 214/2186 | 1 (Ref.) | 1 (Ref.) | 1.00(ref.) | 1 (Ref.) | 1 (Ref.) |
| Q2 | 138/2185 | 0.60(0.48-0.74) | 0.74(0.60-0.92) | 0.72(0.57-0.90) | 0.70(0.56-0.87) | 0.66(0.53-0.83) |
| Q3 | 116/2185 | 0.53(0.42-0.66) | 0.69(0.55-0.88) | 0.67(0.52-0.87) | 0.69(0.54-0.89) | 0.58(0.45-0.76) |
| Q4 | 97/2185 | 0.48(0.38-0.61) | 0.77(0.60-0.99) | 0.72(0.54-0.96) | 0.76(0.57-1.01) | 0.60(0.44-0.81) |
| Q5 | 67/2185 | 0.44(0.33-0.58) | 0.82(0.61-1.10) | 0.74(0.53-1.03) | 0.78(0.56-1.08) | 0.56(0.39-0.79) |
| *p* for trend |  | <0.001 | 0.017 | 0.015 | 0.015 | <0.001 |
| CER |  |  |  |  |  |  |
| Q1 | 78/2185 | 1 (Ref.) | 1 (Ref.) | 1.00(ref.) | 1 (Ref.) | 1 (Ref.) |
| Q2 | 104/2185 | 1.01(0.75-1.36) | 0.79(0.58-1.06) | 0.82(0.60-1.11) | 0.81(0.60-1.10) | 0.86(0.64-1.17) |
| Q3 | 93/2186 | 0.85(0.62-1.14) | 0.60(0.44-0.81) | 0.65(0.47-0.89) | 0.64(0.46-0.88) | 0.75(0.54-1.04) |
| Q4 | 134/2185 | 1.12(0.85-1.49) | 0.70(0.52-0.94) | 0.74(0.54-1.01) | 0.70(0.51-0.96) | 0.93(0.67-1.30) |
| Q5 | 223/2185 | 2.02(1.56-2.62) | 1.01(0.76-1.34) | 1.08(0.78-1.50) | 1.03(0.74-1.43) | 1.50(1.05-2.13) |
| *p* for trend |  | <0.001 | <0.001 | <0.001 | <0.001 | <0.001 |

^a^ Data was HR (95%CI). METs, individual’s income, intake of energy, fruit, vegetable, whole-grain, saturated fat, monounsaturated fat and polyunsaturated fat, urbanization index were used mean values during the survey.

^b^ Model 1 was crude model.

^c^ Model 2 was further adjustment for demographic covariates including age, sex, smoking status, alcohol status, mean METs (total metabolic equivalents), education levels, mean individual’s income.

^d^ Model 3 was further adjustment for nutritional covariates including mean intake of energy, fruit, vegetable, whole-grain, saturated fatty acid, monounsaturated fatty acid and polyunsaturated fatty acid.

^e^ Model 4 was further adjustment for mean BMI.

^f^ Model 5 was further adjustment for social environment covariates including mean urbanization index, province, medical insurance.

^g^ FER, energy provided by dietary fat; CER, energy provided by dietary carbohydrate.
